# Decreasing Greenhouse Gas Emissions from the Municipal Solid Waste Sector in Chinese Cites

**DOI:** 10.1021/acs.est.4c00408

**Published:** 2024-06-14

**Authors:** Shijun Ma, Nana Deng, Chuan Zhao, Peng Wang, Chuanbin Zhou, Chuanlian Sun, Dabo Guan, Zhaohua Wang, Jing Meng

**Affiliations:** ‡ The Bartlett School of Sustainable Construction, University College London, London WC1E 6BT, United Kingdom; § School of Economics, Beijing Institute of Technology, Beijng 100081, People’s Republic of China; ∥ Digital Economy and Policy Intelligentization Key Laboratory of Ministry of Industry and Information Technology, Beijing 100081, People’s Republic of China; ⊥ Graduate School of Environmental Studies, Tohoku University, Sendai, Miyagi 980-8579, Japan; # Key Laboratory of Urban Environment and Health, Institute of Urban Environment, Chinese Academy of Sciences, Xiamen, Fujian 361021, People’s Republic of China; ∇ Stake Key Laboratory of Urban and Regional Ecology, Research Center for Eco-Environmental Sciences, Chinese Academy of Sciences, Beijing 100085, People’s Republic of China; ○ College of Resources and Environment, University of Chinese Academy of Sciences, Beijing 101408, People’s Republic of China; ◆ Department of Earth System Science, Ministry of Education Key Laboratory for Earth System Modeling, Institute for Global Change Studies, Tsinghua University, Beijing 100084, People’s Republic of China

**Keywords:** municipal solid waste, disposal, greenhouse gases, city, scenarios

## Abstract

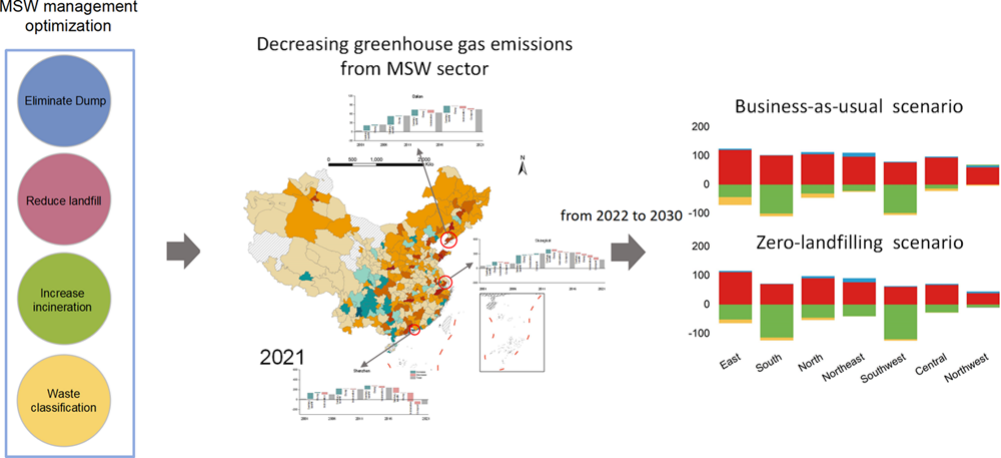

Municipal solid waste (MSW) management systems play a crucial role in greenhouse gas (GHG) emissions in China. Although the government has implemented many policies to improve the MSW management system, the impact of these improvements on city-level GHG emission reduction remains largely unexplored. This study conducted a comprehensive analysis of both direct and downstream GHG emissions from the MSW sector, encompassing sanitary landfill, dump, incineration, and biological treatment, across 352 Chinese cities from 2001 to 2021 by adopting inventory methods recommended by the Intergovernmental Panel on Climate Change (IPCC). The results reveal that (1) GHG emissions from the MSW sector in China peaked at 70.6 Tg of CO_2_ equiv in 2018, followed by a significant decline to 47.6 Tg of CO_2_ equiv in 2021, (2) cities with the highest GHG emission reduction benefits in the MSW sector were historical emission hotspots over the past 2 decades, and (3) with the potential achievement of zero-landfilling policy by 2030, an additional reduction of 203.7 Tg of CO_2_ equiv is projected, with the emission reduction focus toward cities in South China (21.9%), Northeast China (17.8%), and Southwest China (17.3%). This study highlights that, even without explicit emission reduction targets for the MSW sector, the improvements of this sector have significantly reduced GHG emissions in China.

## Abstract 2

Improvements in municipal solid waste management systems enable deep methane emission reductions in China without any emission reduction targets

## Introduction

China, as one of the largest annual emitters of greenhouse gas (GHG) emissions,^1^ has pledged to peak its carbon emissions before 2030 and aims to achieve carbon neutrality before 2060.^2^ This requires fast decarbonization and emission reductions of all sectors. Solid waste treatment is the fourth largest source of emissions, accounting for 3% of the total GHG emissions.^3,4^ Driven by socioeconomic development and population expansion, the amount of municipal solid waste (MSW) generation has surged by 30 times since the 1950s, paralleling a rapid rise in GHG emissions from this sector in China.^5^ The GHG emissions from MSW disposal primarily comprise carbon dioxide (CO_2_) emissions from incineration, methane (CH_4_) emissions emitted by landfills, and few amounts of methane and nitrous oxide (N_2_O) emissions in biological treatment.^6,7^ Notably, the MSW sector is one of the priorities of the Global Methane Pledge at the 26th United Nations Climate Change Conference of the Parties^8,9^ because of its great reduction potential for methane emissions.

The formulation and implementation of policies play a crucial role in the sustainable development of energy and the environment.^10,11^ For example, Bertolino et al. argue that Brazil can attract more investments in the renewable energy sector and foster sustainable development by establishing stable and consistent policies.^12^ Barra and Falcone have identified a positive and significant impact of institutional quality on environmental efficiency.^13^ Specifically, an Italian case shows that policy strategies can effectively improve the municipal solid waste management (MSWM) systems through social innovation, technological innovation, and scientific and technological cooperation among stakeholders, thus reducing environmental impacts.^14^ China has promulgated a series of policies to promote the improvement of MSWM systems over the past 2 decades.

Since the 2000s, following the revision of the “Law on Solid Waste” and the implementation of the “MSW Classification Management Approach”, the proportion of MSW harmless treatment in China has rapidly increased from less than 60% in 2000 to nearly 100% at present.^15,16^ Furthermore, during the 13th Five-Year Plan (2016–2020), the Chinese government set goals for a diversified waste treatment strategy,^17^ including reducing landfill use, eliminating illegal dumps, increasing waste incineration, and enhancing recycling. In 2019, the Ministry of Ecology and Environment launched the “zero-waste city” plan, designating 10 cities to spearhead endeavors aimed at minimizing waste generation, promoting recycling practices, and enhancing MSW management.^18^ Moreover, MSW classification policies have been implemented in cities, such as Shanghai and Beijing, to increase the efficiency of incineration by separating out recyclable and hazardous waste, ensuring that the MSW sent to incinerators is more suitable for incineration.^19,20^ Accurately qualifying and forecasting the GHG emission reduction benefits of the MSW sector resulting from these policies can provide a reference for MSWM in other developing countries and assist the policymakers to design future GHG mitigation strategies.

Recent studies have extensively analyzed the GHG emissions from the MSW sector in China and its GHG emission mitigation potential at the national and regional levels. For instance, Yang et al. explored the GHG emissions of the MSW sector in Ningbo in 2018 and proposed that the implementation of the “zero-waste city” policy could increase the GHG emission reduction of the MSWM system by 2.8 times by 2025.^16^ Case studies in Shanghai and Qingdao demonstrated that the MSW classification could significantly decrease the GHG emission intensity.^20,21^ Other research also explored the driving factors of GHG emissions from the MSW sector in China.^5,22−27^ However, these research mainly addresses the direct emissions from the MSW sector, often neglecting the emission reduction benefits of byproducts during MSW disposals, such as incineration with energy recovery, anaerobic digestion, and composting.^28^ Consequently, the emission reduction potential of recent MSWM optimization policies may be substantially underestimated. Our study broadens the GHG emission accounting boundary of the MSW sector by considering both direct and downstream GHG emissions of different MSW disposal methods. This approach will contribute to a more comprehensive understanding of GHG emissions in this sector. Addressing this gap is crucial for accurately evaluating and enhancing the role of the MSW sector for emission reduction efforts in China.

In this study, we estimate the GHG emissions of the MSW sector in China, adhering to the Intergovernmental Panel on Climate Change (IPCC) approach,^29^ with a city-level analysis encompassing 352 cities from 2001 to 2021. We detail the calculation process for GHG emissions from four kinds of disposal methods, including sanitary landfills, dumps, incineration, and biological treatment. Additionally, we employ a counterfactual analysis to assess historical emission reductions in the MSW sector and develop two scenarios, “business as usual” and “zero landfilling”, to forecast future GHG emission reductions from 2022 to 2030. Details can be found in the Methods and Data section. Our findings reveal significant temporal and spatial variations in GHG emissions across 352 cities, illustrating how advancements in the MSW sector have notably contributed to reducing GHG emissions. Furthermore, our projections regarding future GHG emissions for the MSW sector can assist policymakers in defining future emission pathways and designing effective GHG mitigation strategies.

## Methods and Data

### System Boundary and Quantification Procedures

This study systematically considers the accounting of GHG emissions from various disposal methods, including sanitary landfills, dumps, incineration, and biological treatment, in 352 Chinese cities from 2001 to 2021. The GHG emission calculations include both direct GHG emissions caused by MSW disposal activities as well as GHG emission reduction benefits resulting from the energy recovery or resource utilization of byproducts generated during the MSW disposal process. We estimate direct GHG emissions for each type of MSW disposal method using inventory methods recommended by IPCC.^29^ Furthermore, we also calculate the GHG emission reduction benefits from incineration power generation, replacing coal-fired power generation, as well as the GHG emission reduction benefits from composting, replacing chemical fertilizer. Additionally, for anaerobic digestion, we calculate the GHG emission reduction benefits from biogas power generation, replacing coal-fired power generation.

In addition, all parameters in the study are obtained from multiple data sources, including statistical yearbooks, numerous reports, and literature. The data source information is summarized in Table S1 of the Supporting Information.

#### GHG Emissions of Sanitary Landfills and Dumps

The main GHG emissions in landfills are methane and carbon dioxide. However, carbon dioxide emissions generated through biomass degradation are often excluded from current GHG emission accounting frameworks.^30^ Therefore, our study focuses solely on estimating methane emissions from landfills, utilizing the first-order decomposition model recommended by IPCC, as shown in eq 1.^31^ The combined GHG emissions from sanitary landfills and dumps are calculated by eq 2
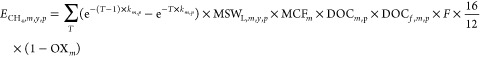
1where *E*_CH_4_,*m*,*y*,*p*_ represents the amount of methane emissions in landfill type *m* for city *p* in year *y* (Tg a^–1^/million tons a^–1^), *T* is the time when the MSW is deposited into landfills, the constant *k*_*m*,*p*_ denotes the methane production rate in landfill type *m* for city *p*, which is calculated using eq S1 of the Supporting Information, MSW_L,*m*,*y*,*p*_ refers to the amount of MSW disposed in landfill type *m* for city *p* in year *y* (Tg a^–1^), MCF_*m*_ is the methane correction factor of landfill type *m*,^28^ DOC_*m*,*p*_ represents the biodegradable organic carbon content in MSW in landfill type *m* for city *p*, which can be calculated by eq S2 of the Supporting Information, DOC_*f*,*m*,*p*_ is the fraction of DOC that can be oxidized in landfill type *m* for city *p*, which can be calculated by eq S3 of the Supporting Information, *F* represents the volume fraction of methane in landfill gas, set to 0.5,^24^ and OX_*m*_ is the oxidation factor in landfill type *m*. Landfill types include sanitary landfills and dumps. Equations S1–S3 are detailed in section 1.1 of the Supporting Information
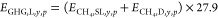
2where *E*_GHG,L,*y*,*p*_ represents the amount of GHG emissions in all landfills for city *p* in year *y* (Tg a^–1^) and *E*_CH_4_,SL,*y*,*p*_ and *E*_CH_4_,D,*y*,*p*_ represent the amount of methane emissions in sanitary landfills and dumps for city *p* in year *y* (Tg a^–1^), respectively. The warming potential of methane is 27.9 times that of carbon dioxide, and the warming potential of nitrous oxide is 273 times that of carbon dioxide.^28^

#### GHG Emissions of Incineration

The direct GHG emissions from incineration are mainly carbon dioxide. The incineration of organic carbon is not included in the accounting of direct GHG emissions. We account only for GHG emissions from the incineration of fossil carbon. On the basis of the material balance theory, the GHG emissions from incineration can be calculated according to eq 3
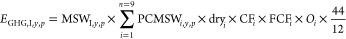
3where *E*_GHG,I,*y*,*p*_ is the amount of direct GHG emissions of incineration in city *p* in year *y* (Tg a^–1^), MSW_I,*y*,*p*_ is the amount of MSW disposed of through incineration in city *p* in year *y* (Tg a^–1^), PCMSW_*i*,*y*,*p*_ is physical composition of municipal solid waste in city *p* in year *y*, PCMSW_*i*_ includes the organic fraction, ash and stone, paper, plastic and rubber, textile, wood, metal, glass, and others, dry_*i*_ is the proportion of combustible dry matter in PCMSW_*i*_, and CF_*i*_, FCF_*i*_, and *O*_*i*_ are the fraction of carbon, the fraction of fossil carbon, and oxidation rate of combustible dry matter in PCMSW_*i*_, respectively.

Given that incineration plants basically generate electricity for energy recovery in China,^32^ we calculate the GHG emission reduction benefits brought about by replacing coal-fired power generation with incineration power generation and include it in the final calculation of incineration GHG emissions, as shown in eq 4
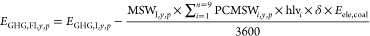
4where *E*_GHG,FI,*y*,*p*_ is the amount of final GHG emissions of incineration in city *p* in year *y* (Tg a^–1^), δ is the electricity conversion efficiency for incineration, hlv_*i*_ is the heat value of PCMSW_*i*_ (kJ/kg), and *E*_ele,coal_ is the GHG emission generated by coal-fired power generation (kg/kWh).^33^

#### GHG Emissions of Biological Treatment

The biological treatment of organic fractions in China mainly comes from composting and anaerobic digestion. The main GHG emissions from composting are methane and nitrous oxide, while the emissions from anaerobic digestion are escaped methane. Li et al. pointed out that 76.1% of organic waste in China is anaerobically digested, and the rest is composted. We use this ratio to calculate direct GHG emissions from biological treatment in Chinese cities. See eqs 5–7 for details^34^
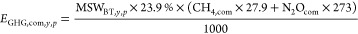
5
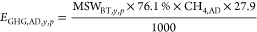
6
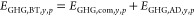
7where *E*_GHG,BT,*y*,*p*_, *E*_GHG,com,*y*,*p*_, and *E*_GHG,AD,*y*,*p*_ represent the amount of direct GHG emissions of biological treatment, composting, and anaerobic digestion in city *p* in year *y*, respectively (Tg a^–1^), MSW_BT,*y*,*p*_ is the amount of MSW disposed of through biological treatment in city *p* in year *y* (Tg a^–1^), CH_4,com_ and N_2_O_com_ are the direct GHG emissions of composting of 1 ton of MSW (kg/t), and CH_4,AD_ is the direct GHG emissions of anaerobic digestion of 1 ton of MSW (kg/t).

In addition, composting provides fertilizer, while methane from anaerobic digestion is mostly captured to generate electricity.^3^ Therefore, in this study, the GHG emission reduction benefits as a result of the replacement of nitrogen fertilizer by compost and the replacement of coal-fired power generation by electricity generated through anaerobic digestion are calculated and included in the final GHG emission accounting for biological treatment. See eqs 8–10 for details
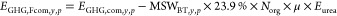
8
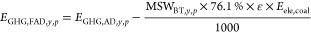
9
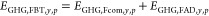
10where *E*_GHG,FBT,*y*,*p*_, *E*_GHG,Fcom,*y*,*p*_, and *E*_GHG,FAD,*y*,*p*_ represent the amount of final GHG emissions of biological treatment, composting, and anaerobic digestion in city *p* in year *y*, respectively (Tg a^–1^), *N*_org_ is the nitrogen content of the organic fraction, μ is the nitrogen content of urea fertilizer, *E*_urea_ is GHG emissions for producing 1 kg of urea fertilizer (kg/kg), and ε is the electricity conversion efficiency of anaerobic digestion (kWh/t).

The GHG emissions of the total MSW sector in Chinese cities are further calculated, namely, eq 11.
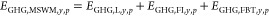
11

### Scenario Settings for Mitigation Pathways through the Optimization of MSWM Systems

#### Forecast of MSW Generation for Chinese Cities

The MSW generation in cities is closely related to socioeconomic indicators, especially urban population (POP) and per capita gross domestic product (PCGDP).^35^ Thus, for the 292 Chinese cities with historical POP and PCGDP data, we use the multiple linear regression model (eq 12) to predict the amount of MSW generated from 2022 to 2030, while for the other 60 cities, the autoregressive integrated moving average model is used; see details in eq 13. Using the above method has good effects for the prediction of MSW generation for Chinese cities. The predicted data are very close to the real historical data, demonstrating an impressive *R*_2_ value of 0.89 (Figure S1 of the Supporting Information). Besides, the forecasts of POP and PCGDP for each city from 2022 to 2030 are detailed in section 1.2 of the Supporting Information
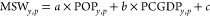
12where MSW_*y*,*p*_ is MSW generation in city *p* in year *y*, POP_*y*,*p*_ is the urban population in city *p* in year *y*, and PCGDP_*y*,*p*_ is total gross national product per capita in city *p* in year *y*
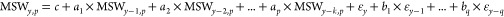
13where ε_*y*_ is the random error in year *y*, *a*, *b*, and *c* are the coefficients, respectively, and *k* and *q* are integers that are often referred to as autoregressive and moving average, respectively.

We set up two scenarios, namely, an actual scenario and a counterfactual scenario, to reflect the GHG emission reduction benefits brought about by the optimization of the MSWM system based on historical years (2001–2021). Then, on the basis of the predicted MSW generation, two scenarios, including the business-as-usual scenario and the zero-landfilling scenario, are set up to explore the GHG emission reduction benefits brought by achieving zero waste to landfills in the future.

#### Actual Scenario

It describes the GHG emissions caused by the real situation of the MSWM system in Chinese cities from 2001 to 2021. All MSW disposal data comes from the *China Urban and Rural Construction Statistical Yearbook*.^36^

#### Counterfactual Scenario

It describes that the MSW disposal ability of all Chinese cities remains at the level in 2001, and the GHG emissions from the MSW sector are calculated from 2001 to 2021. This scenario is to compare to the actual scenario and shows the GHG emission reduction caused by the optimization of the MSWM system in China in the past 20 years, including the elimination of illegal dumps, decreasing landfilling rate, waste classification, and increased waste incineration. Under this scenario, the amount of MSW by disposal methods in each city is calculated according to eq 14
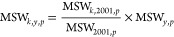
14where MSW_*k*,2001,*p*_ and MSW_*k*,*y*,*p*_ are the amount of MSW treated by method *k* in a certain city *p* in 2001 and year *y* and MSW_2001,*p*_ is the amount of MSW generation in a certain city *p* in 2001. Treatment methods include sanitary landfills, dumps, incineration, and biological treatment. *y* is from 2001 to 2021.

#### Business-as-Usual Scenario

It assumes that MSW disposal ability in Chinese cities will remain at the level of 2021 in the future. Under this scenario, the amount of MSW by disposal methods in each city is calculated according to eq 15
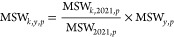
15where MSW_*k*,2021,*p*_ and MSW_*k*,*y*,*p*_ are the amount of MSW treated by method *k* in a certain city *p* in 2021 and year *y* and MSW_2021,*p*_ is the amount of MSW generation in a certain city *p* in 2021. *y* is from 2022 to 2030.

#### Zero-Landfilling Scenario

It assumes that Chinese cities will continue to implement the zero-waste-landfill policy and accelerate the development of incineration from 2022 to 2030.^37^ In comparison to the business-as-usual scenario, with the socioeconomic development, the amount of MSW incineration in each city follows the logistic curve and grows close to the amount of MSW generation as the year changes.^38^ See eq 16 for details. Our model has good prediction results for waste incineration. The predicted data align with the real historical data, exhibiting a *R*_2_ value of 0.95 (Figure S2 of the Supporting Information).

It is worth noting that, as of 2021, there are still 93 cities in China with an incineration rate of 0, and nearly 70% of them are distributed in Northwest China, Southwest China, and Central China. It is assumed that the incineration rate in these cities will reach the average level of each province in the future. Besides, the amount of other disposal methods will be weighted and distributed according to the MSW disposal characteristics of each city in 2021, as shown in eq 17
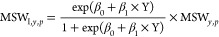
16
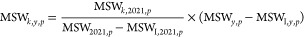
17where MSW_SL,2021,*p*_, MSW_D,2021,*p*_, MSW_I,2021,*p*_, and MSW_BT,2021,*p*_ are the amounts of MSW treated by sanitary landfills, dumps, incineration, and biological treatment in a certain city *p* in 2021, respectively, β_0_ and β_1_ are constants, and *Y* denotes the year.

## Results

### GHG Emissions from the MSW Sector over Time

**Figure 1:**
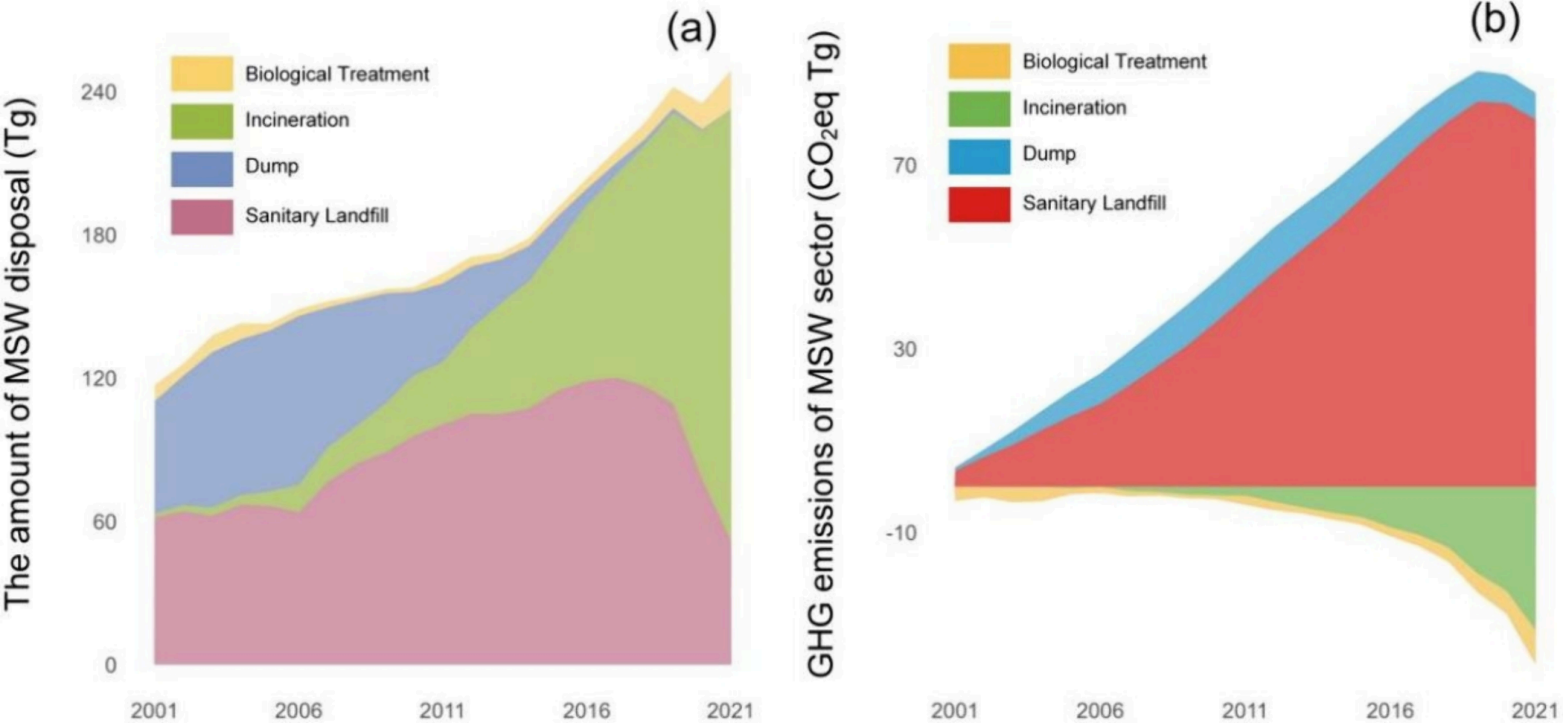
Disposal amount and GHG emissions of the MSW sector in China from 2001 to 2021: (a) disposal amount and (b) GHG emissions.

Over the past 2 decades, we have witnessed a radical change in the main MSW disposal methods in China, shifting from sanitary landfills and dumps to incineration (Figure 1a). The proportion of dumps dropped sharply from 40.2% in 2001 to just 0.1% in 2021, while incineration experienced a remarkable surge from 1.4% in 2001 to 72.5% in 2021. Particularly, incineration increased by nearly 56.7% in the past decade alone. The utilization of sanitary landfills fluctuated, maintaining between 51.5 and 61.5% until 2018, peaking in 2012, before sharply declining to 20.9% by 2021. Since 2011, this fundamental shift of MSW disposal methods in China has been primarily driven by a series of policy implementations, including the elimination of informal dump sites outlined in the 13th Five-Year Plans as well as the enforcement of the zero-waste cities and zero-waste-to-landfill policies.^7^

The shift of MSW disposal methods in China over the past 20 years also led to changes in GHG emissions from the MSW sector, which can be mainly divided into three stages over this period (Figure 1b and Figure S3 of the Supporting Information). The first stage witnessed rapid growth, with the GHG emissions from the MSW sector escalating from 1.3 Tg of CO_2_ equiv (million tons) in 2001 to 47.0 Tg of CO_2_ equiv in 2011 as a result of the use of sanitary landfills and dumps as the main disposal methods. During this period, the GHG emissions from sanitary landfills and dumps increased from 3.4 and 0.7 Tg of CO_2_ equiv in 2001 to 41.3 and 9.5 Tg of CO_2_ equiv in 2011, respectively. In 2001, the reduction in GHG emissions in the MSW sector in China was primarily due to biological treatment, with GHG emissions of −3.1 Tg of CO_2_ equiv, while the GHG emissions of incineration were only 0.02 Tg of CO_2_ equiv. By 2011, GHG emissions from incineration were basically equivalent to those from biomass treatment at −2.0 Tg of CO_2_ equiv.

The second stage, from 2012 to 2017, was characterized by slow growth in GHG emissions from the MSW sector in China. During this period, emissions only increased from 51.5 to 69.3 Tg of CO_2_ equiv. The rise in this period was mainly due to the increase in methane emissions from sanitary landfills, which increased from 46.8 Tg of CO_2_ equiv in 2012 to 74.5 Tg of CO_2_ equiv in 2017. In contrast, the GHG emissions from dumps in 2017 were only 78.9% of those in 2012, primarily attributed to the decreasing proportion of MSW being disposed in dumps. Meanwhile, the reduction in GHG emissions from incineration in 2017 reached 3.2 times that of 2012. The third stage, beginning in 2018, marked a period of rapid decline in GHG emissions from the MSW sector in China. This downward trend saw emissions drop from 70.6 Tg of CO_2_ equiv in 2018 to 47.6 Tg of CO_2_ equiv in 2021. During this period, methane emissions from sanitary landfills peaked in 2019 at 83.8 Tg of CO_2_ equiv. The reduction in GHG emissions from incineration witnessed a significant variation from −13.3 to −31.3 Tg of CO_2_ equiv, while that from biological treatment changed from −3.1 to −7.5 Tg of CO_2_ equiv. This notable reduction in GHG emissions can be attributed to the growing prevalence of incineration facilities and the implementation of food waste separation strategies.

### Heterogeneity of GHG Emissions in the MSW Sector across Chinese Cities

**Figure 2:**
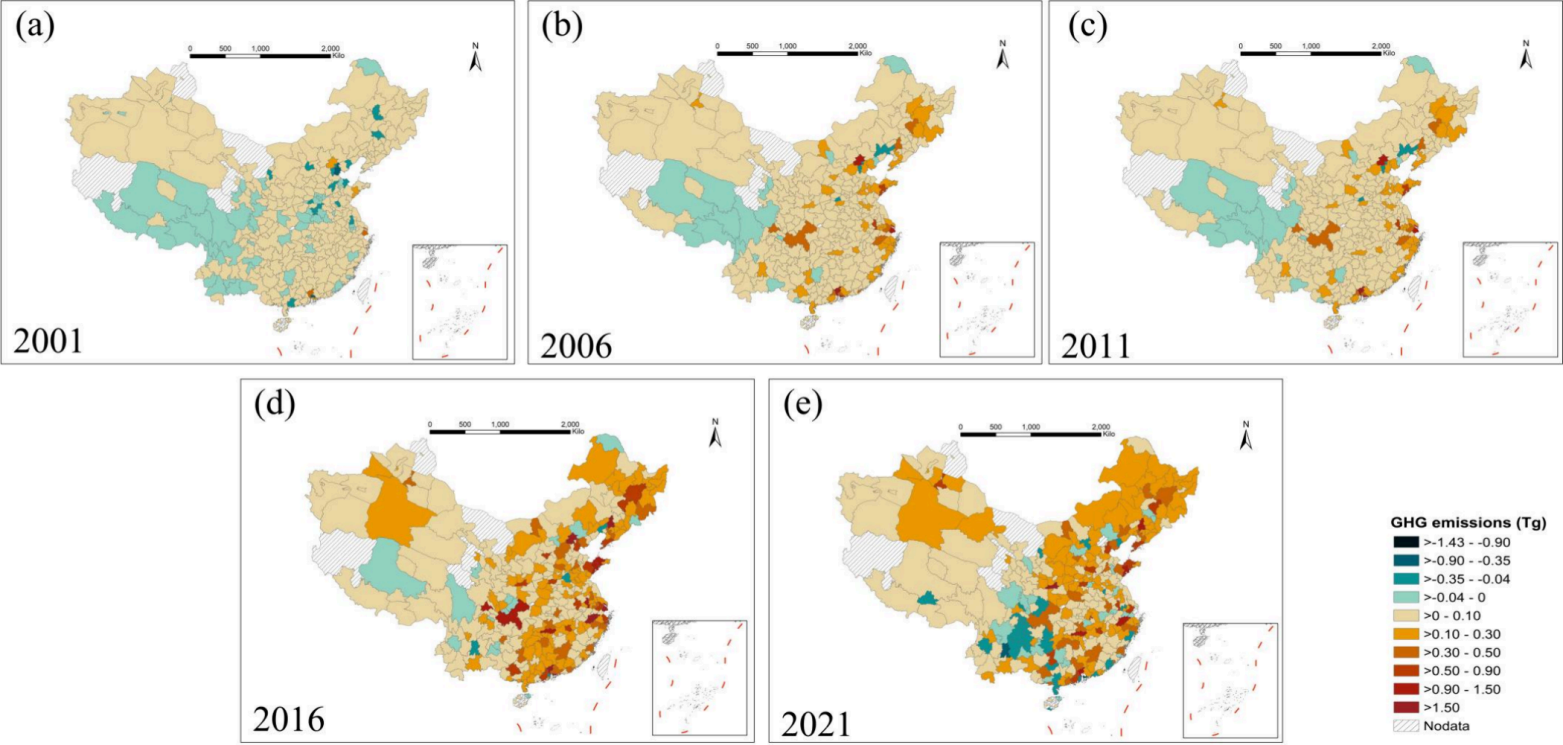
Spatial distribution of GHG emissions from the MSW sector in China: (a) 2001, (b) 2006, (c) 2011, (d) 2016, and (e) 2021.

**Figure 3:**
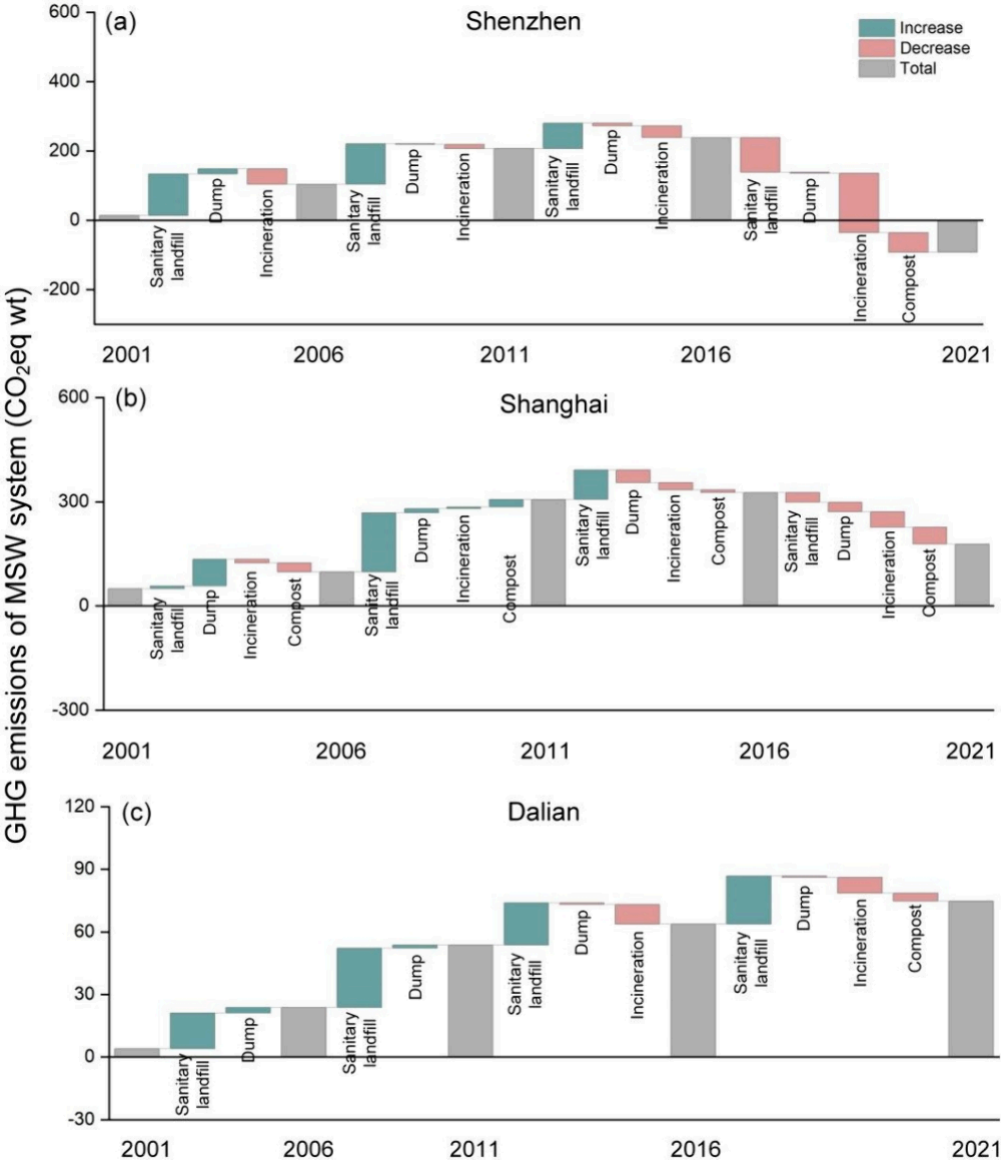
GHG emission patterns of the MSW sector in specific cities from 2001 to 2021: (a) Shenzhen, (b) Shanghai, and (c) Dalian.

The GHG emissions from the MSW sector in 352 Chinese cities from 2001 to 2021 are further explored (Figure 2). We found that cities with high GHG emission reduction benefits from their MSW sector were historical emission hotspots. From 2001 to 2016, most Chinese cities witnessed an upward trajectory in GHG emissions from their MSW sector, with emission hotspots predominantly in provincial capital cities and southern coastal cities. In 2016, the top 10 cities contributing to GHG emissions in the MSW sector were Guangzhou, Shanghai, Beijing, Shenzhen, Shenyang, Hangzhou, Chongqing, Qingdao, Changsha, and Nanjing, collectively emitting GHG emissions of 21.0 Tg of CO_2_ equiv, accounting for 31.7% of the total (Figure 2d). By 2021, the GHG emissions from the MSW sector of these cities dropped significantly (Figure 2d). They emitted only 10.8 Tg of CO_2_ equiv, which was 51.5% of their 2016 emissions. Meanwhile, their emissions accounted for only 22.7% of the total emissions from the Chinese MSW sector in 2021. The 10 cities with the highest GHG emissions from the MSW sector have achieved significant emission reductions (Figure 3 and Figure S4 of the Supporting Information).

Among these, some cities became carbon-negative in the MSW sector by 2021 (Figure 3a). For example, the GHG emissions of the MSW sector in Shenzhen increased from 0.2 Tg of CO_2_ equiv in 2001 to 2.5 Tg of CO_2_ equiv in 2016 and dropped to −0.8 Tg of CO_2_ equiv in 2021. This is mainly because such a city has basically achieved zero landfilling and promoted an organic waste separation policy.^7^ In 2021, the amount of incineration and biological treatment accounted for 83.8 and 16.0% of the total MSW generation, and only 0.2% went to sanitary landfills, resulting in negative GHG emissions of the MSW sector in Shenzhen. Although the GHG emissions from the MSW sector in most of the top 10 cities followed a downward trend from 2016 to 2021, their emissions still remained above zero by 2021 (Figure 3b). For example, the GHG emissions of the MSW sector in Shanghai increased from 0.5 Tg of CO_2_ equiv in 2001 to 3.4 Tg of CO_2_ equiv in 2016 and then dropped to 2.1 Tg of CO_2_ equiv in 2021. Despite actively promoting zero-waste-landfill policies, a small fraction of MSW still entered into sanitary landfills in such cities. Additionally, the substantial historical stock of organic waste in landfills continuously releases significant GHGs, maintaining positive GHG emissions of the MSW sector.

In contrast, GHG emissions from the MSW sector continued to rise in some northern cities, especially in Northeast China and North China, primarily because landfills remain the predominant MSW disposal method (Figure 3c). For example, the GHG emissions from the MSW sector in Dalian increased from 0.04 Tg of CO_2_ equiv in 2001 to 0.8 Tg of CO_2_ equiv in 2021, with the sanitary landfills accounting for over 50% of waste generation throughout the past 2 decades.

### Decreasing GHG Emissions from the MSW Sector in China

**Figure 4:**
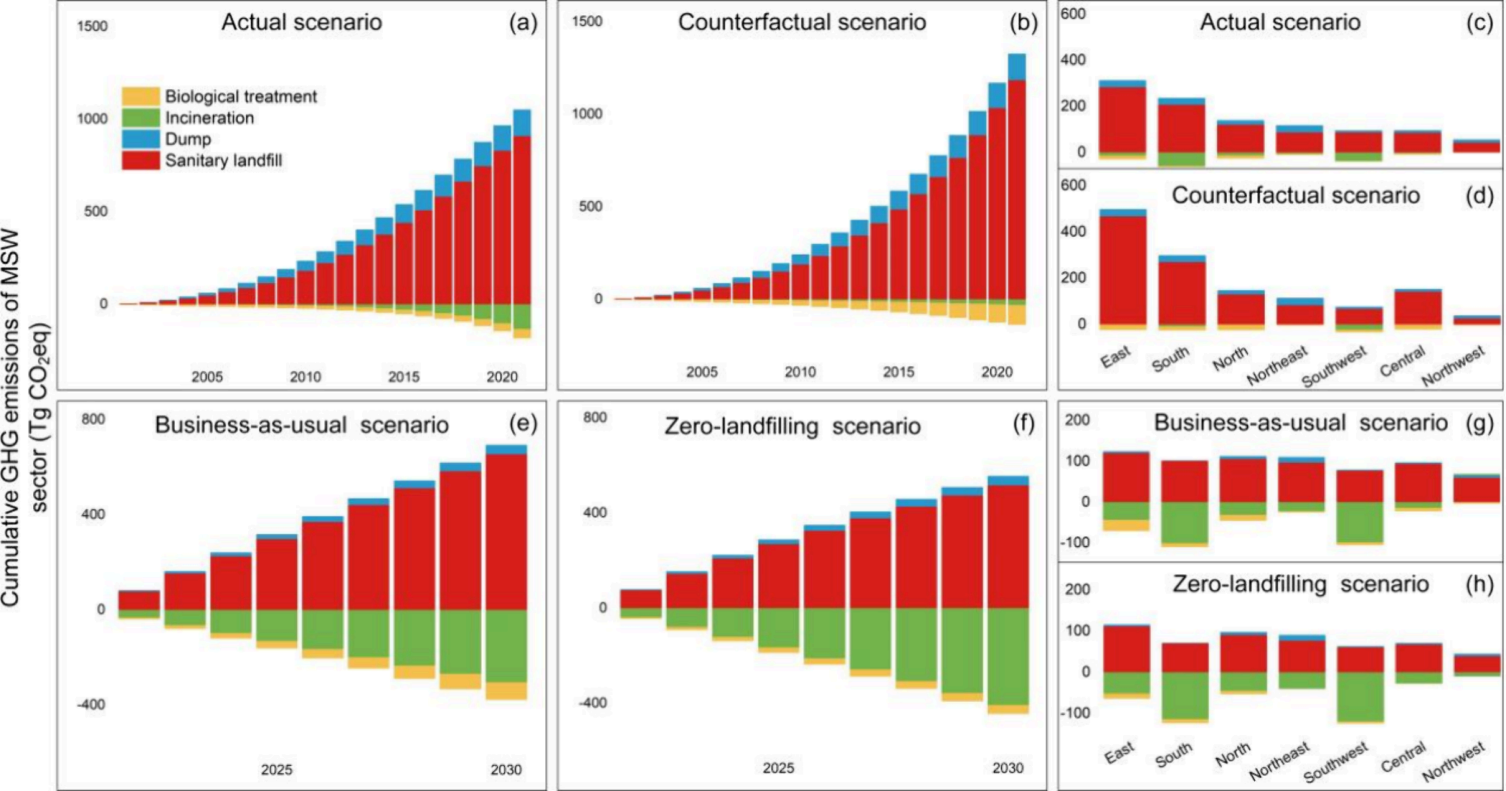
Cumulative GHG emissions of the MSW sector from 2001 to 2030: (a and b) cumulative GHG emissions of the MSW sector in China from 2001 to 2021 (a) under the actual scenario and (b) under the counterfactual scenario, (c and d) cumulative GHG emissions of the MSW sector by region from 2001 to 2021 (c) under the actual scenario and (d) under the counterfactual scenario, (e and f) cumulative GHG emissions of the MSW sector in China from 2022 to 2030 under (e) the business-as-usual scenario and (f) under the zero-landfill scenario, and (g and h) cumulative GHG emissions of the MSW sector by region from 2022 to 2030 (g) under the business-as-usual scenario and (h) under the zero-landfill scenario.

To further analyze the impact of the improvement from the MSWM system on GHG emission reduction in China, we set up two comparative scenarios based on historical and future years, respectively. For historical years, the actual scenario shows the real GHG emissions in Chinese cities from 2001 to 2021, while the counterfactual scenario assumes that the waste disposal level of cities in 2001 will be retained until 2021 (panels a–d of Figure 4). For 2022–2030, we set up two future scenarios to explore the impact of the continuous improvement of the MSWM system in China on further GHG emission reduction. The business-as-usual scenario assumes that from 2022 to 2030, the MSW disposal level of cities will remain the same as in 2021. The zero-landfill scenario assumes that Chinese cities will persist in implementing the zero-waste-landfill policy and expedite the advancement of incineration from 2022 to 2030 (panels e–h of Figure 4).

The results show that, under the actual scenario, the cumulative GHG emissions from the MSW sector in China from 2001 to 2021 amounted to 868.1 Tg of CO_2_ equiv. If the MSW disposal level remained at the level of 2001, that is, under the counterfactual scenario, the cumulative GHG emissions of the MSW sector in China increased by 320.5 Tg of CO_2_ equiv, which was 6.8 times the GHG emissions of the MSW sector in 2021. In addition, the GHG emissions of the MSW sector under the actual scenario are only 32.9% of those under the counterfactual scenario in 2021 (panels a and b of Figure 4). Interestingly, there are significant regional differences in emission reductions from the MSW sector in China. In comparison to the counterfactual scenario, the actual scenario saw cumulative emission reductions of 191.2 Tg of CO_2_ equiv in East China and 100.9 Tg of CO_2_ equiv in South China over the past 2 decades, while emissions in Southwest China and Northwest China even increased by 11.8 and 19.3 Tg of CO_2_ equiv, respectively. This disparity is mainly due to differences in the solid waste management level between the regions. Developed regions were more proactive in reducing landfills and promoting waste incineration, whereas less developed areas exhibit the opposite trend. For example, the landfill rate in East China dropped from 94.8% in 2001 to 4.1% in 2021, whereas the landfill rate in Northwest China remained high at 49.4% by 2021 (panels c and d of Figure 4). However, this also means that there is greater GHG emission reduction space in the MSW sector of the Northwest and Southwest regions.

We further analyzed the GHG emission reductions of the MSW sector in China from 2022 to 2030 under different scenarios. Under the business-as-usual scenario, which assumes the continuation of MSWM practices in 2021, the cumulative GHG emissions of the MSW sector are projected to 314.9 Tg of CO_2_ equiv from 2022 to 2030. However, if Chinese cities continue to implement zero-waste-to-landfill policies, that is, under the zero-landfilling scenario, the cumulative GHG emissions from the MSW sector in 2022–2030 are only 35.3% of those in the business-as-usual scenario (panels e and f of Figure 4). Remarkably, under the zero-landfilling scenario, the MSW sector in China is projected to achieve negative carbon emissions by 2029. From 2022 to 2030, the majority of emission reductions in the MSW sector are expected in South China, Northeast China, and Southwest China, accounting for 21.9, 17.8, and 17.3% of the total cumulative GHG emission reductions, respectively (panels g and h of Figure 4).

## Discussion

Our study assessed the GHG emissions from the MSW sector across 352 Chinese cities between 2001 and 2021, specifically focusing on sanitary landfills, dumping, incineration, and biological treatment. Unlike previous studies that primarily focused on direct GHG emissions or a single disposal method at the national or regional level, our research offers a comprehensive analysis of GHG emissions from the MSW sector in Chinese cities. Moreover, we also highlighted the past and future GHG emission reduction benefits, owing to the improvement of MSWM systems in China. The environmental benefits that we have identified could provide quantitative evidence to accelerate the implementation of supportive MSWM optimization policies in the future. In addition, our estimates and scenarios of GHG emissions from the MSW sector in China are subject to uncertainties and limitations (see detailed description in section 1.3 and Figure S5 of the Supporting Information).

The optimization of the MSWM systems in China led to significant reductions in GHG emissions from 2001 to 2021. We observed a rapid growth trend in the GHG emissions from the MSW sector in China before 2016, which is consistent to the existing studies.^5^ However, a noteworthy shift occurred post-2016, with GHG emissions from the MSW sector exhibiting a slow growth rate and even a notable downward trend. This change can be largely attributed to the adoption of incineration as the main MSW disposal methods during the “13th Five-Year Plan” in China.^24^ In incineration plants with energy recovery, MSW is combusted to produce electricity and heat, thus effectively replacing coal, which is a more emission-intensive fuel. This process decreases methane emissions from landfills and increases energy efficiency, resulting in significant GHG emission reduction.^39^ Given the rapid increase in the number of MSW incineration plants, it is imperative to require these facilities to adopt CO_2_ control technologies, which will further enhance the GHG emission reduction of the MSW sector in China.^40^

In addition, the reduction in GHG emissions from biological treatment has also increased slightly over the past 20 years. The biological treatment, such as composting and anaerobic digestion, significantly reduces GHG emissions by transforming organic waste into valuable resources. Composting converts organic waste into fertilizer, reducing the reliance on GHG-intensive synthetic fertilizers, while anaerobic digestion produces biogas, replacing coal, which can be used for electricity generation. Moreover, studies suggest that MSW classification policies can effectively improve the separation of organic waste, thus promoting the development of biological treatment of MSW.^41,42^ By the end of 2022, 297 prefecture-level cities have piloted or implemented MSW classification policies.^43^ However, as a result of multiple reasons, such as the policy implementation gap from grassroot-level governments and strong obstacles from incineration companies, MSW classification has been stagnant.^44^ Determining MSWM priorities, refining MSW classification policies and standards, and improving recycling infrastructure will pave the way for the future advancement of MSW classification, thereby achieving greater GHG emission reductions in the MSW sector.

The spatial heterogeneity in the characteristics of MSW treatment in Chinese cities leads to variations in GHG emission trends and reduction potential within the MSW sector.^45^ On the basis of the GHG emissions from the MSW sector and local realities, each city should implement targeted mitigation measures. Our results indicate that, over the past 20 years, the GHG emission reductions from the MSW sector mainly came from East China and South China, especially from provincial capital cities and developed coastal cities. These cities are historical emission hotspots from the MSW sector. However, as a result of the large deployment of incineration in recent years, they also became the focus of GHG emission reductions. For cities that have basically achieved zero waste to landfill, such as Shenzhen, governments should encourage and support the adoption of modern carbon capture and storage (CCS) technologies at incineration facilities to further reduce GHG emissions.^46,47^ For cities that have a large amount of stored MSW in landfills, such as Beijing and Shanghai, in addition to using CCS technologies, integrated proactive measures should be taken, including landfill mining, illegal dump elimination, and landfill gas collection system installation.^48^

In regions such as Northeast China and Southwest China, landfilling remains the primary method of waste disposal. Thus, in comparison to other regions, such as East China, GHG emissions from the MSW sector in these regions decreased more slowly or even increased. For these cities, like Dalian, it is crucial for the government to offer incentives to encourage landfill operators to retrofit their facilities for landfill gas collection and further utilization.^49^ The governments of these cities should also provide financial support for new technologies of MSW disposal, implement stricter regulatory frameworks for MSW management, and promote public and corporate awareness of MSW classification.^41,42,46^ This will facilitate the transition from landfills to incineration or resource recovery. It is noteworthy that the potential emission reduction space will concentrate in Northeast China and Southwest China from 2021 to 2030. However, the great GHG reduction potential in these undeveloped regions will bring more financial burdens.^3,50^ Thus, to achieve more drastic GHG emission reduction targets by 2030, optimization policies in MSWM systems, particularly in Northeast China and Southwest China, should be intensified across various aspects, including technological development, consumer behavior, and institutional coordination.^51^

In China, the disposal methods should further shift from dumps and landfills to incineration and resource recovery. Recovering energy from waste is an important strategy to make MSW management more sustainable.^52^ Moreover, educational and awareness campaigns can foster environmental values, leading to reduced waste generation at the source, such as lessening food and plastic waste.^53^ This initiative should be complemented by the advancement of MSW classification and recycling measures to enable more efficient resource recovery and processing.^54^ The deployment of advanced GHG emission reduction technologies is also critical in minimizing GHG emissions from MSW sectors in China.^46^ Moreover, the exchange of knowledge and technologies between cities, especially from those with successful GHG emission reduction strategies to those falling behind, is imperative for nationwide GHG emission reduction progress.^43^ Such a unified and robust approach is vital for achieving MSWM goals in China and contributing to global warming mitigation efforts.

## Supporting Information Available

Discussions of estimation methods of methane emissions in landfills, prediction methods of urban population (POP) and per capita gross domestic product, and uncertainty analysis, tables of data sources of all parameters, methane production rate coefficient, degradable organic carbon, decomposable biodegradable organic carbon, methane oxidation factor, key parameters for CO_2_ emissions, and geographical regions of China, and figures of comparison of the predicted value and actual value for per capita gross domestic product, urban population, MSW generation and incineration, GHG emissions of the MSW sector in China, the GHG emission patterns of the MSW sector in the top 10 cities with the highest GHG emissions of the MSW sector, and uncertainty analysis (PDF)
