# Supplementary material for: Decreasing Greenhouse Gas Emissions from the Municipal Solid Waste Sector in Chinese Cites
Source: Environ Sci Technol. 2024 Jun 14;58(26):11342–51. doi: 10.1021/acs.est.4c00408 (PMC11223490; doi:10.1021/acs.est.4c00408)
Supplement: Supplementary file 1 — es4c00408_si_001.pdf [file es4c00408_si_001.pdf]

Supplementary Information

Decreasing greenhouse gas emission from municipal solid waste management system in Chinese cites

Shijun Ma[a,i], Nana Deng[b,c,i], Chuan Zhao[d], Peng Wang[e], Chuanbin Zhou[f,g], Chuanlian Sun[f,g], Dabo Guan[a,h], Zhaohua Wang[b,c*], Jing Meng[a*]

Author affiliations:

a. The Bartlett School of Sustainable Construction, University College London, London, WC1E 6BT, UK;

b. School of Economics, Beijing Institute of Technology, Beijing, China, 100081；

c. Digital Economy and Policy Intelligentization Key Laboratory of Ministry of Industry and Information Technology，Beijing, China, 100081;

d. Graduate School of Environmental Studies, Tohoku University, Sendai, 980-8579, Japan;

e. Key Lab of Urban Environment and Health, Institute of Urban Environment, Chinese Academy of Sciences, Xiamen 361021, China;

f. Stake Key Laboratory of Urban and Regional Ecology, Research Center for Eco-Environmental Sciences, Chinese Academy of Sciences, Beijing 100085, China;

g. College of Resources and Environment, University of Chinese Academy of Sciences, Beijing 101408, China;

h. Department of Earth System Science, Ministry of Education Key Laboratory for Earth System Modeling, Institute for Global Change Studies, Tsinghua University, Beijing 100084, China.

i. These authors contributed equally

Number of pages: 14

Number of Tables: 9

Number of Figures: 6

Content

Supplementary Information S1
Supplementary text...S3
1.1 The calculation of some parameters in the estimation model of methane emissions in landfills S3
1.2 The prediction of urban population (POP) and per capita gross domestic product (PCGDP) S3
1.3 Uncertainty Analysis...S3
Supplementary tables S4
Table S1 the data sources of all parameters for accounting greenhouse gas emissions of municipal solid waste sector in Chinese cities S4
Table S2 Methane production rate coefficient S6
Table S3 Degradable organic carbon...S7
Table S4 Decomposable biodegradable organic carbon S7
Table S5 Methane oxidation factor...S7
Table S6 The crucial key parameter value for CO_2 emissions for MSW incineration S7
Table S7 The heating value of each component of MSW S7
Table S8 Electricity generation efficiency for incineration by region.....................................S8
Table S9 Geographical regions of China...S8
Supplementary figures S9
Figure S1 the comparison of predicted value and actual value for MSW generation (n=7392)S9
Figure S2 the comparison of predicted value and actual value for MSW incineration (n=7392) S10
Figure S3 GHG emissions of MSW sector in China from 2001 to 2021. ....S11
Figure S4 the GHG emissions patterns of MSW sector in the top ten cities with the highest GHG emissions of MSW sector from 2001 to 2021...S12
Figure S5 the uncertainty analysis of GHG emissions of MSW sector from 2001 to 2030 under the actual scenario and zero-landfilling scenario.. ....S13
Figure S6 the comparison of predicted value and actual value for per capita gross domestic product (a) and urban population (b). (n=9928) S14
Reference S14

Supplementary text

1.1 The calculation of some parameters in the estimation model of methane emissions in landfills

In the FOD model recommended by the IPCC, the methane production rate constant (k), degradable organic carbon (DOC) and fraction of DOC that can decompose (DOCf) were calculated by Equations (S1)-(S3).

$$k_{m,p} = k_a \times A_{m,p} + k_b \times B_{m,p} + k_c \times C_{m,p} + k_d \times D_{m,p} \quad \text{Equation (S1)}$$

$$DOC_{m,p} = DOC_a \times A_{m,p} + DOC_b \times B_{m,p} + DOC_c \times C_{m,p} + DOC_d \times D_{m,p} \quad \text{Equation (S2)}$$

$$DOCf_{m,p} = DOCf_a \times A_{m,p} + DOCf_b \times B_{m,p} + DOCf_c \times C_{m,p} + DOCf_c \times D_{m,p} \quad \text{Equation (S3)}$$

where $A_{m,p}$-$D_{m,p}$ represent the proportions of organic fractions, paper, textile and wood in municipal solid waste (MSW) in a prefecture-level city p in a geographical region r, respectively; the methane production rate for each component is detailed in Table S2; the DOC of each component in MSW is shown in Table S3; and the DOCf of each component in MSW is shown in Table S4.

1.2 The prediction of urban population (POP) and per capita gross domestic product (PCGDP)

The POP and PCGDP data from 2022 to 2030 are the basis for predicting MSW generation in Chinese cities. First, we collected as much historical POP and PCGRP data as possible in Chinese cities (1988~2021). Based on this dataset, for each Chinese city, the time series model, the autoregressive integrated moving average model, is constructed to predict future POP and PCGDP. See Equation (S4) and (S5) for details. We forecast the POP and PCGRP of each city from 1988 to 2021 and compared it with the real data. The R^2 was 0.85 and 0.98 respectively, which means that the time series model has a good prediction effect for POP and PCGRP (Figure S5).

$$POP_{y,p} = \mathrm{c} + \mathrm{a}_1 \times POP_{y-1,p} + \mathrm{a}_2 \times POP_{y-2,p} + \ldots + \mathrm{a}_\mathrm{p} \times POP_{y-k,p} + \varepsilon_y + \mathrm{b}_1 \times \varepsilon_{y-1} + \ldots + \mathrm{b}_\mathrm{q} \times \varepsilon_{y-q} \quad \text{Equation (S4)}$$

$$PCGDP_{y,p} = \mathrm{c} + \mathrm{a}_1 \times PCGDP_{y-1,p} + \mathrm{a}_2 \times PCGDP_{y-2,p} + \ldots + \mathrm{a}_\mathrm{p} \times PCGDP_{y-k,p} + \varepsilon_y + \mathrm{b}_1 \times \varepsilon_{y-1} + \ldots + \mathrm{b}_\mathrm{q} \times \varepsilon_{y-q} \quad \text{Equation (S5)}$$

Where, POP and PCGRP are urban population and per capita gross domestic product; ε_y is the random error in year y; a and b are the coefficients, respectively; k and q are integers that are often referred to as autoregressive and moving average, respectively.

1.3 Uncertainty Analysis

In our study, we mainly perform predictions on the amount of MSW generation and disposal, especially incineration, at the city level. The accuracy of these data predictions will bring huge uncertainties to GHG emission estimates from the MSW sector at the city scale.

First, in order to predict the amount of MSW generation and incineration at the city level from 2022 to 2030, we construct the multiple linear regression or time series models. We compare the actual historical generation and incineration data from 2001 to 2021 with the values fitted by the models, and found that R^2 are 0.89 and 0.95 respectively (Figure S1 and S2). This reflects the robustness of the forecast to a certain extent.

Furthermore, to more fully assess the uncertainty in the results, we perform a comprehensive analysis of GHG emission estimates from the MSW sector at the city scale. Following the uncertainty analysis method shown by Tong et al. (2018)[1], we conduct 10000 Monte Carlo analyzes by changing the key variable parameters(the amount of MSW generation and incineration), to quantify the uncertainty of GHG emissions from MSW sector in each city in China. For the amount of MSW generation and incineration in historical years, we assume that

these data are normally distributed, with the coefficient of variation (CV) of 10%[2]. For predicted data from 2022 to 2030, we use the data uncertainty calculated by multiple linear regression or time series models.

The uncertainty analysis on GHG emissions from the MSW sector in Chinese cities from 2001 to 2021 is conducted. The results show that the GHG emission accounting for incineration and biological treatment has strong uncertainty, with a CV of 0.10 (95% confidence interval (CI)), while the uncertainty in GHG emission accounting for sanitary landfills and dumps is relatively small, with a CV of only 0.05 (95% CI). This results in the CV of GHG emissions from the MSW sector of Chinese cities fluctuating between 0.08 and 0.14 (95% CI) from 2001 to 2021 (Figure S5(a)).

In addition, we also conduct an uncertainty analysis on the GHG emissions of the MSW sector in Chinese cities from 2022 to 2030 under the zero-landfilling scenario. Because the amount of MSW generation and disposal is derived from predictions, the accounting results are highly uncertain. The CVs of GHG emission accounting for sanitary landfills, dumps, incineration and biological treatment are 0.13~0.16, 0.04~0.05, 0.68~1.37 and -6.25~30.08 respectively. This results in the CV of GHG emissions from the MSW sector at the city level fluctuating between 1.59 and 8.22 (95% CI) from 2001 to 2021 (Figure S5(b)). We estimate that GHG emissions from the MSW sector in China increase from 1.02 CO_2eq Tg (0.36 CO_2eq Tg~1.68 CO_2eq Tg) in 2001 to 70.23 CO_2eq Tg (67.00 CO_2eq Tg~73.46 CO_2eq Tg) in 2018 and then decrease to -6.97 CO_2eq Tg (-57.57 CO_2eq Tg~43.64 CO_2eq Tg) in 2030 (Figure S5(c)).

Supplementary tables

Table S1 the data sources of all parameters for accounting greenhouse gas emissions of municipal solid waste sector in Chinese cities

Parameters	abbreviation	formula	references
the amount of MSW disposed of in landfill type m for city p in year y	$MSW_{L, m, y, p}$		China Urban Construction Statistical Yearbook (2001-2021)[3]
the amount of waste incineration in city p in year y	$MSW_{I,y,p}$		China Urban Construction Statistical Yearbook (2001-2021)[3]
the amount of biological treatment in city p in year y	$MSW_{BT,p}$		China Urban Construction Statistical Yearbook (2001-2021)[3]
the constant of methane production rate in landfill type m for city p	$k_{m,p}$	Equation (S1)	
the methane production rate for each component	k_a-k_d	Table S2	Fei et al. (2019)[4]

methane correction factor of landfill type m	MCF_m	1.0 for sanitary landfills and 0.6 for dumps	Cai et al. (2018)[5]
the biodegradable organic carbon content in MSW in landfill type m for city p	$DOC_{m,p}$	Equation (S2)	
the DOC of each component in MSW	DOC_a-DOC_d	Table S3	Gao et al. (2007)[6]
the fraction of DOC that can be oxidized in landfill type m for city p	$DOC_{f, m, p}$	Equation (S3)	
fraction of DOC that can decompose in MSW	$DOCf_a$-$DOCf_d$	Table S4	Chen and Xue (2013)[7]
the volume fraction of methane in landfill gas	F	0.5	Fei et al. (2019)[4]
the oxidation factor in landfill type m	OX_m	Table S5	Cai et al. (2018)[5]
the physical composition of municipal solid waste in city p in year y	$PCMSW_{i,y,p}$		Ma et al. (2020)[8]
the proportion of combustible dry matter in $PCMSW_i$	Dry_i	Table S6	Bian et al. (2022)[9]
the fraction of carbon in $PCMSW_i$	CF_i	Table S6	Bian et al. (2022)[9]
the fraction of fossil carbon in $PCMSW_i$	FCF_i	Table S6	Bian et al. (2022)[9]
oxidation rate of combustible dry matter in $PCMSW_i$	O_i	1.0	Bian et al. (2022)[9]
the heat value of PCMSW i	hlv_i	Table S7	Zhou et al. (2022)[9]
the electricity conversion efficiency for incineration by region	δ	Table S8	Cheng et al. (2007)[10]; Zhou et al. (2022)[11]; Tang et al. (2013)[12]; Liu et al. (2022)[13]
the GHG emission generated by	$E_{Ele,coal}$	1.345 kg/kWh	Ecoinvent[14]

coal-fired power generation			
the direct methane emissions of composting of 1 ton MSW	$CH4_{com}$	0.004 tCH_4/t	Yang et al. (2023)[15]
the direct N_2O emissions of composting of 1 ton MSW	$N2O_{com}$	0.00024 tCH_4/t	Yang et al. (2023)[15]
the direct methane emissions of anaerobic digestion of 1 ton MSW	$CH4_{AD}$	0.0008 tCH_4/kt	Yang et al. (2023)[15]
nitrogen content of urea fertilizer	μ	0.45	Ma et al. (2023)[16]
nitrogen content of organic fraction	N_{org}	0.108	Ma et al. (2023)[16]
GHG emissions for producing 1kg of urea fertilizer	N_{org}	5.91 kg/lg	Ecoinvent[14]
electricity conversion efficiency of anaerobic digestion	ε	150 kWh/t	Yang et al. (2023)[15]
urban population in city p in year y	$POP_{y,p}$		China urban Statistical Yearbook (2002-2022)[17]
total gross national product in city p in year y	$PCGDP_{y,p}$		China urban Statistical Yearbook (2002-2022)[17]

Table S2 Methane production rate coefficient

Type of Waste		Climate Zone<br>Temperate (MAT≤20℃)		Tropics (MAT>20℃)	
		Dry (MAP/PET< 1)	Wet (MAP/PET< 1)	Dry (MAP<1000 mm)	Wet (MAP≥1000 mm)
Slowly Degrading Waste	Paper/Textile	0.04	0.06	0.045	0.07
	Wood	0.02	0.03	0.025	0.035
Rapidly Degrading Waste	Organic waste	0.06	0.185	0.085	0.4

Table S3 Degradable organic carbon

Component	Northeast, North and Northwest China %	Southwest, Central-South, East China %
Paper	28.53	23.34
Wood	35.51	21.07
Textile	27.68	32.71
Kitchen waste	10.19	4.27

Table S4 Decomposable biodegradable organic carbon

Component	DOCf
Organic waste	0.8
Wood	0.5
Plastic and Rubber	0.0
Paper	0.5
Textile	0.2

Table S5 Methane oxidation factor

Landfill type	Northwest China	North, Northeast, Central and East China	South and Southwest China
sanitary landfill	0	0	0
dumps	0.15	0.2	0.3

Table S6 The crucial key parameter value for CO_2 emissions for MSW incineration

MSW component	Dry matter content in % of wet weight	Total carbon content in % of dry weight	Fossil carbon fraction in % of total carbon
Organic waste	40	38	0
Ash and stone	100	20.3	100
Plastic and rubber	100	75	100
Paper	90	46	1
Wood	85	50	0
Textile	80	50	20
Metal	100	21	100
Glass	100	0.3	100
Others	100	20.3	100

Table S7 The heating value of each component of MSW

Physical composition of municipal solid waste	Heat value (kJ/kg)
Organic fraction	4650
Ash and stone	2935
Paper	16525
Plastic and rubber	31998
Textile	17450

Wood	6510
Glass	140
Metal	700
Others	2935

Table S8 Electricity generation efficiency for incineration by region

Region	Northeast, Northwest, North[10]	Central, East[11]	Southwest, South[12, 13]
Electricity generation efficiency	0.146	0.124	0.198

Table S9 Geographical regions of China

Geographical region	Province
China	Central China, East China, North China, Northeast China, Northwest China, South China, Southwest China
Northeast China	Heilongjiang, Jilin, Liaoning, Northeast Hebei (Chengde, Qinhuangdao), East Inner Mongolia (Xilinguole League, Chifeng, Tongliao, Hinggan League, Hulun Buir)
Northwest China	Shaanxi, Gansu, Qinghai, Ningxia, Xinjiang, West Inner Mongolia (Alxa League, Bayan Nur, Wuhai)
Central-South China	Henan, Hubei, Hunan, Guangdong, Guangxi, Hainan
East China	Shanghai, Jiangsu, Zhejiang, Anhui, Jiangxi, Shandong, Fujian, Taiwan
North China	Beijing, Tianjin, Central and South Hebei, Shanxi, Central Inner Mongolia (Hohhot, Ulanqab, Baotou, Ordos)
Northeast China	Heilongjiang, Jilin, Liaoning, Northeast Hebei (Chengde, Qinhuangdao), East Inner Mongolia (Xilinguole League, Chifeng, Tongliao, Hinggan League, Hulun Buir)

Supplementary figures

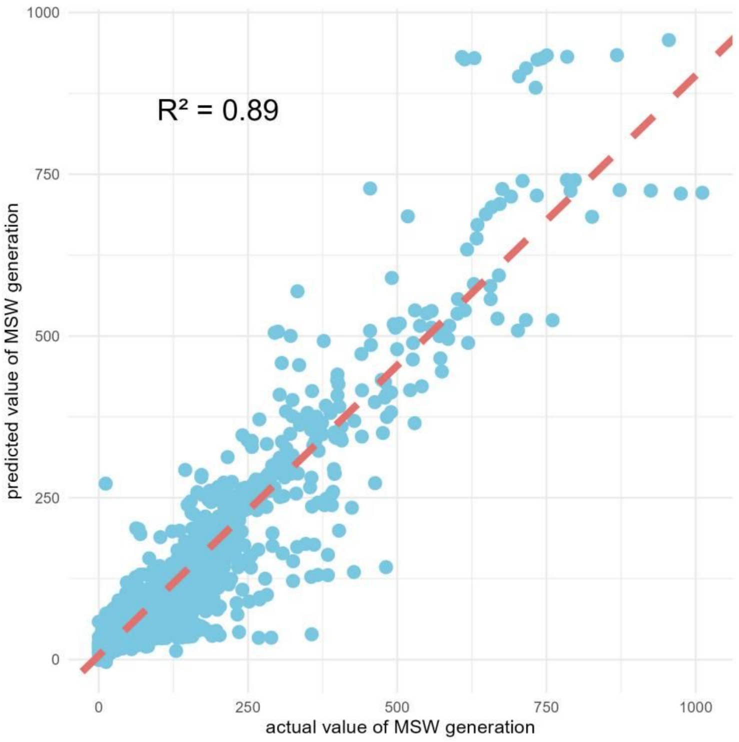

Figure S1 the comparison of predicted value and actual value for MSW generation (n=7392)

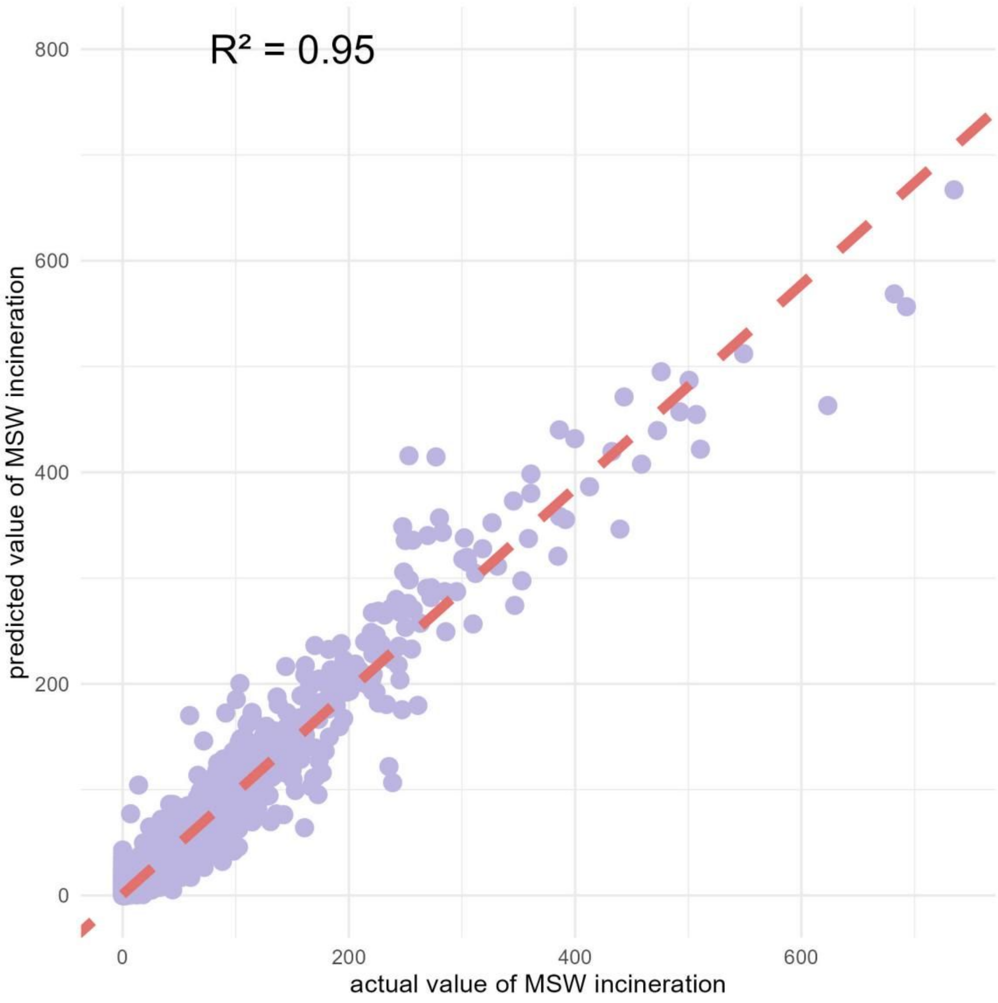

Figure S2 the comparison of predicted value and actual value for MSW incineration (n=7392)

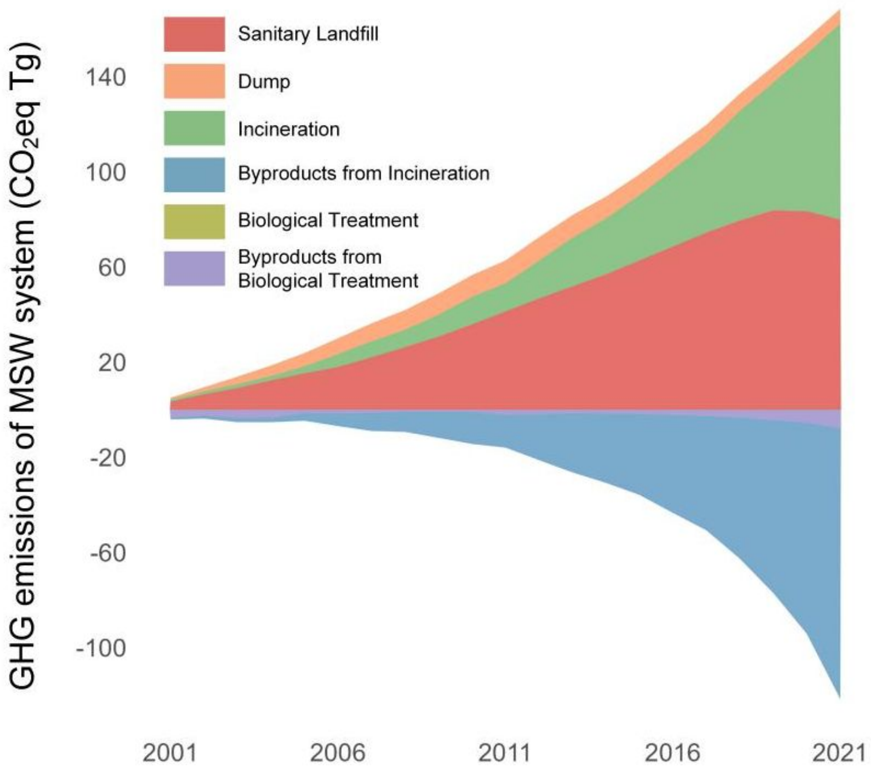

Figure S3 GHG emissions of MSW sector in China from 2001 to 2021.

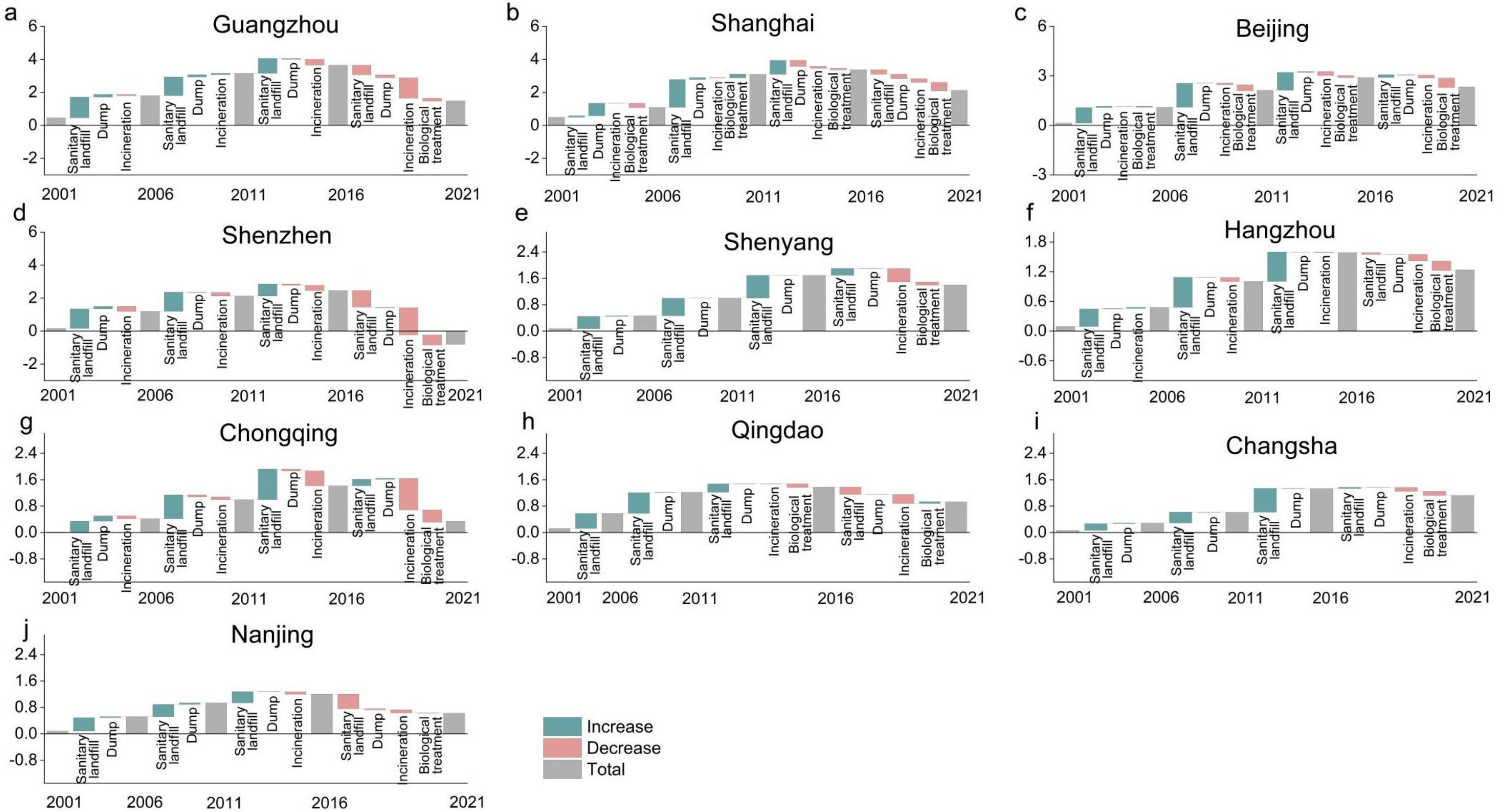

Figure S4 the GHG emissions patterns of MSW sector in the top ten cities with the highest GHG emissions of MSW sector from 2001 to 2021. (a) Guangzhou; (b) Shanghai; (c) Beijing; (d) Shenzhen; (e) Shenyang; (f) Hangzhou; (g) Chongqing; (h) Qingdao; (i) Changsha; (j) Nanjing

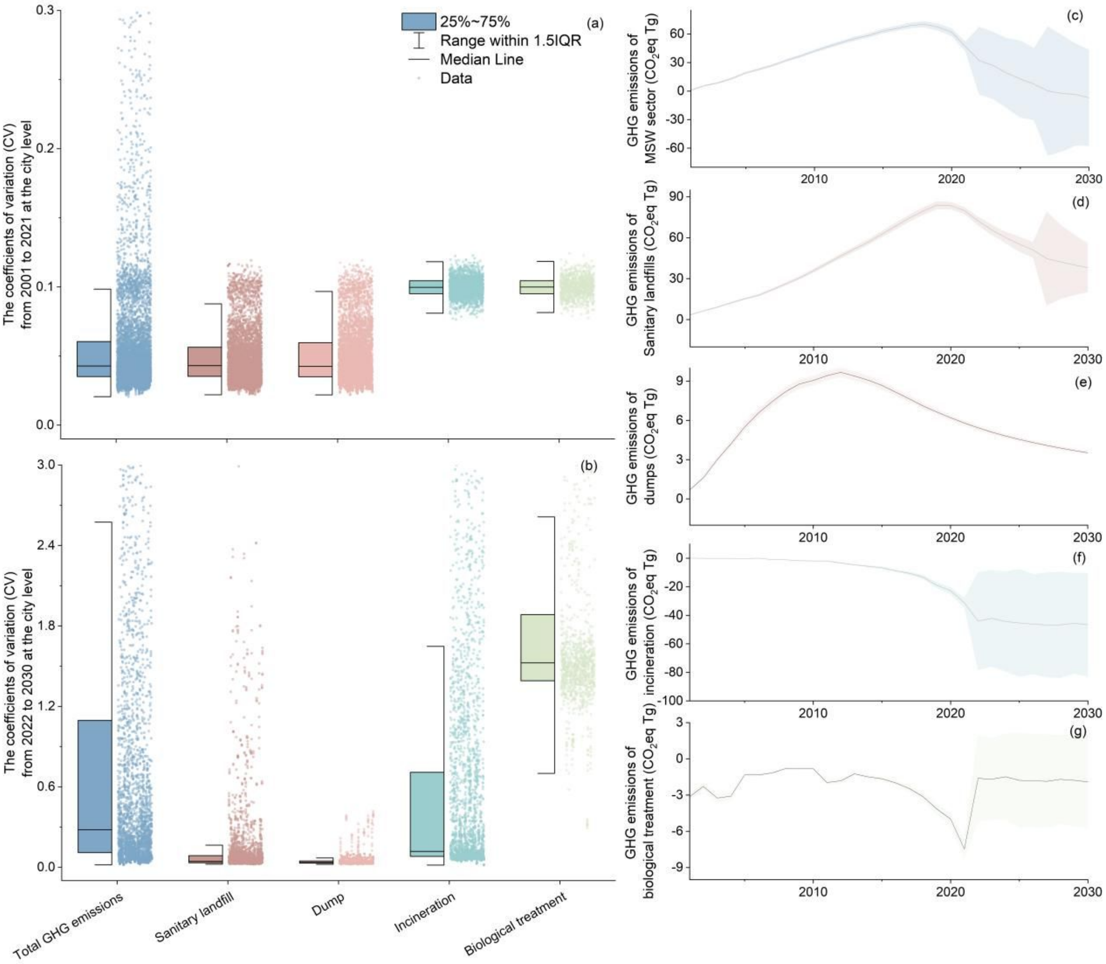

Figure S5 the uncertainty analysis of GHG emissions of MSW sector from 2001 to 2030 under the actual scenario and zero-landfilling scenario. (a) the coefficients of variation from 2001 to 2021 at the city level; (b) the coefficients of variation from 2002 to 2030 at the city level; the uncertainty of GHG emissions of MSW sector (c), sanitary landfills (d), dumps (e), incineration (f), and biological treatment (g) in China from 2001 to 2030.

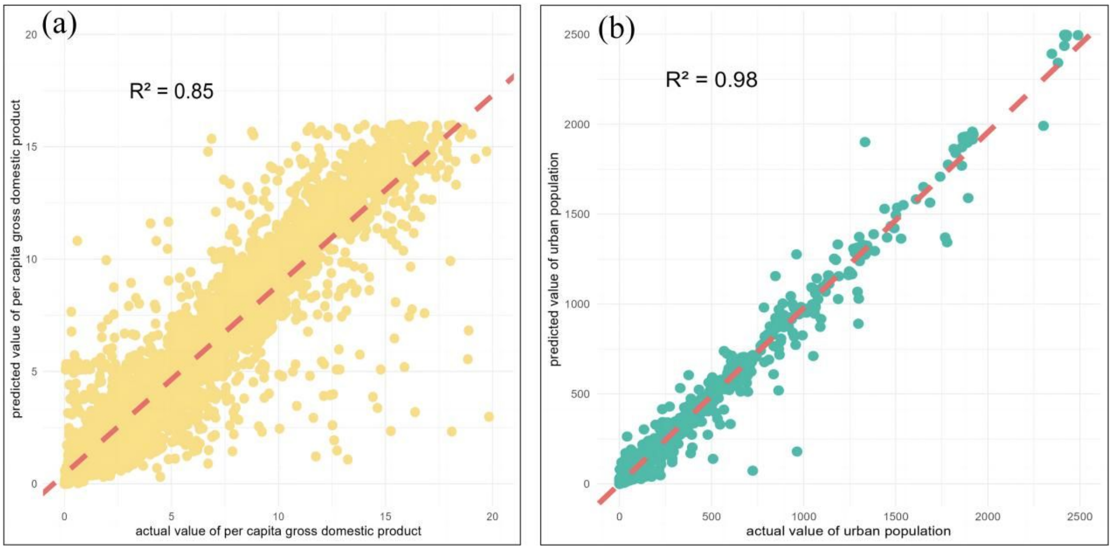

Figure S6 the comparison of predicted value and actual value for per capita gross domestic product (a) and urban population (b). (n=9928)

Reference

(1) Tong, D.; Zhang, Q.; Davis, S. J.; Liu, F.; Zheng, B.; Geng, G.; Xue, T.; Li, M.; Hong, C.; Lu, Z. Targeted emission reductions from global super-polluting power plant units. *Nat. Sustain.* **2018**, *1* (1), 59-68.

(2) Lei, T.; Wang, D.; Yu, X.; Ma, S.; Zhao, W.; Cui, C.; Meng, J.; Tao, S.; Guan, D. Global iron and steel plant CO2 emissions and carbon-neutrality pathways. *Nature* **2023**, *622* (7983), 514-520.

(3) MOHURD. *China Urban and Rural Construction Statistical Yearbook*; Ministry of Housing and Urban-Rural Development of the People's Republic of China, 2001-2021.

(4) Fei, F.; Wen, Z.; De Clercq, D. Spatio-temporal estimation of landfill gas energy potential: A case study in China. *Renew. Sust. Energ. Rev.* **2019**, *103*, 217-226.

(5) Cai, B.; Lou, Z.; Wang, J.; Geng, Y.; Sarkis, J.; Liu, J.; Gao, Q. CH4 mitigation potentials from China landfills and related environmental co-benefits. *Sci. Adv.* **2018**, *4* (7), eaar8400.

(6) Gao Q; Du W; Lu S; Zhang Z; Zhang E; Wu J; Z, R. The Measurement and Research of Degradable Organic Carbon of Municipal Solid Waste in China (in Chinese with English Abstract). *Research of Environmental Sciences* **2007**, *03*, 10-15.

(7) Chen, Z.; Xue, Q. *Practical technical guide for sanitary landfill engineering of household waste*; 2013.

(8) Ma, S.; Zhou, C.; Chi, C.; Liu, Y.; Yang, G. Estimating physical composition of municipal solid waste in China by applying artificial neural network method. *Environ. Sci. Technol.* **2020**, *54* (15), 9609-9617.

(9) Bian, R.; Chen, J.; Zhang, T.; Gao, C.; Niu, Y.; Sun, Y.; Zhan, M.; Zhao, F.; Zhang, G. Influence of the classification of municipal solid wastes on the reduction of greenhouse gas emissions: A case study of Qingdao City, China. *J. Clean. Prod.* **2022**, *376*, 134275.

(10) Cheng, H.; Zhang, Y.; Meng, A.; Li, Q. Municipal Solid Waste Fueled Power Generation in China: A Case Study of Waste-to-Energy in Changchun City. *Environ. Sci. Technol.* **2007**, *41* (21), 7509-7515.

(11) Zhou, C.; Ma, S.; Yu, X.; Chen, Z.; Liu, J.; Yan, L. A comparison study of bottom‐up and top‐down methods for analyzing the physical composition of municipal solid waste. *J. Ind. Ecol.* **2022**, *26* (1), 240-251.

(12) Tang, Y.; Ma, X.; Lai, Z.; Chen, Y. Energy analysis and environmental impacts of a MSW oxy-fuel incineration power plant in China. *Energ. policy* **2013**, *60*, 132-141.

(13) Liu, Y.; Liao, C.; Tang, Y.; Tang, J.; Sun, Y.; Ma, X. Techno-environmental-economic evaluation of the small-scale municipal solid waste (MSW) gasification-based and incineration-based power generation plants. *J. Taiwan Inst. Chem. E.* **2022**, *141*, 104594.

(14) Frischknecht, R.; Rebitzer, G. The ecoinvent database system: a comprehensive web-based LCA database. *J. Clean. Prod.* **2005**, *13* (13-14), 1337-1343.

(15) Yang, G.; Zhang, Q.; Zhao, Z.; Zhou, C. How does the "Zero-waste City" strategy contribute to carbon footprint reduction in China? *Waste Manage.* **2023**, *156*, 227-235.

(16) Ma, S.; Lu, M.; Yang, G.; Zhi, Y.; Ouyang, Z.; Huang, N.; Zhou, C. Bottom-up accounting of landfills across 346 cities reveals overlooked carbon stocks. **2022**.

(17) NBS. *China urban Statistical Yearbook*; National Bureau of Statistics, 2002-2022.
